# Translating Planetary Health Principles Into Sustainable Primary Care Services

**DOI:** 10.3389/fpubh.2022.931212

**Published:** 2022-07-13

**Authors:** Julia Gonzalez-Holguera, Marie Gaille, Maria del Rio Carral, Julia Steinberger, Joachim Marti, Nolwenn Bühler, Alain Kaufmann, Luca Chiapperino, Ana Maria Vicedo-Cabrera, Joelle Schwarz, Anneliese Depoux, Francesco Panese, Nathalie Chèvre, Nicolas Senn

**Affiliations:** ^1^Competence Centre in Sustainability, University of Lausanne, Lausanne, Switzerland; ^2^Laboratory SPHERE, UMR 7219, University Paris Diderot CNRS, Paris, France; ^3^Institute of Psychology, University of Lausanne, Lausanne, Switzerland; ^4^Institute of Geography and Sustainability, University of Lausanne, Lausanne, Switzerland; ^5^Department of Epidemiology and Health Systems, University Center for Primary Care and Public Health (Unisanté), Lausanne, Switzerland; ^6^STS Lab, Institute of Social Sciences, University of Lausanne, Lausanne, Switzerland; ^7^ColLaboratoire (ColLAB), University of Lausanne, Lausanne, Switzerland; ^8^Institute of Social and Preventive Medicine (ISPM), University of Bern, Bern, Switzerland; ^9^Oeschger Center for Climate Change Research, University of Bern, Bern, Switzerland; ^10^Department of Family Medicine, University Center for Primary Care and Public Health (Unisanté), Lausanne, Switzerland; ^11^Centre Virchow-Villermé and Centre des Politiques de la Terre, Université Paris Cité, Paris, France; ^12^Institute of Social Sciences, University of Lausanne, Lausanne, Switzerland; ^13^Faculty of Geosciences and the Environment, Institute of Earth Surface Dynamics (IDYST), University of Lausanne, Lausanne, Switzerland

**Keywords:** planetary health, primary care, health services, health professionals, interdisciplinary, sustainability

## Abstract

Global anthropogenic environmental degradations such as climate change are increasingly recognized as critical public health issues, on which human beings should urgently act in order to preserve sustainable conditions of living on Earth. “Planetary Health” is a breakthrough concept and emerging research field based on the recognition of the interdependent relationships between living organisms—both human and non-human—and their ecosystems. In that regards, there have been numerous calls by healthcare professionals for a greater recognition and adoption of Planetary Health perspective. At the same time, current Western healthcare systems are facing their limits when it comes to providing affordable, equitable and sustainable healthcare services. Furthermore, while hospital-centrism remains the dominant model of Western health systems, primary care and public health continue to be largely undervalued by policy makers. While healthcare services will have to adapt to the sanitary impacts of environmental degradations, they should also ambition to accompany and accelerate the societal transformations required to re-inscribe the functioning of human societies within planetary boundaries. The entire health system requires profound transformations to achieve this, with obviously a key role for public health. But we argue that the first line of care represented by primary care might also have an important role to play, with its holistic, interdisciplinary, and longitudinal approach to patients, strongly grounded in their living environments and communities. This will require however to redefine the roles, activities and organization of primary care actors to better integrate socio-environmental determinants of health, strengthen interprofessional collaborations, including non-medical collaborations and more generally develop new, environmentally-centered models of care. Furthermore, a planetary health perspective translated in primary care will require the strengthening of synergies between institutions and actors in the field of health and sustainability.

## Introduction

### Anthropogenic Environmental Degradations and Human Health

Human activity is leading to profound worldwide degradation of the ecological systems which support life on Earth (1). The planetary boundaries concept provides a non-negotiable global framework within which human activities can take place while allowing the Earth system as a whole to function sustainably. Many of these thresholds of ecosystem transformations are currently transgressed (climate change, biodiversity integrity, biogeochemical flows of nitrogen and phosphorus and land-system change.) These anthropogenic environmental degradations are increasingly recognized as critical public health issues, on which human beings as individuals and populations should urgently act in order to preserve sustainable conditions of living on Earth (1–6). More frequent heat waves, increased air pollution or the (re)emergence, in previously spared regions, of infectious diseases are all well-known phenomena associated with global warming and natural habitats destructions (7–12). According to predictive models, more frequent wildfires, extreme weather events, rising sea levels, or compromised food security will have dire consequences for the mortality and morbidity of current and future populations (13). Resulting destructions of infrastructures, conflicts, mass migration, and economic loss could contribute to reverse the important gains obtained in life expectancy and global health over the last century. Furthermore, the most vulnerable individuals are likely to be the first to be affected, thus reinforcing health inequalities, the risks and impacts of climate change being *unequally* and *unfairly* distributed across individuals (gender, age), communities (minorities) and countries (low-income) (14, 15). It is thus important to recognize the tight link between human health and worldwide ecosystems preservation. As acknowledged by the World Health Organization (WHO), the current situation and the challenges ahead call for a transformation in the way we interact with the natural environment in order to maintain or even improve human health and wellbeing. If healthcare services will have to adapt to the sanitary impacts of environmental degradations, they should also ambition to accompany and accelerate the societal transformations required to remain within the planetary boundaries. The entire health system requires profound transformations to achieve this, with obviously a key role for public health. But the first line of care represented by primary care might also have an important role to play, being a pivot of the system, covering the entire population with a longitudinal health perspective and where the effect of multiple expositions to environmental degradations can be observed in the first place (16).

Planetary Health is a new transdisciplinary health paradigm, which aims at recognizing the systemic links between human health, socio-cultural environment, non-human living organisms and Earth's natural systems (17–23). This recent concept draws on older initiatives such as One Health or Ecohealth (24). The Planetary Health Alliance defines Planetary Health as “*a solutions-oriented, transdisciplinary field and social movement focused on analyzing and addressing the impacts of human disruptions to Earth's natural systems on human health and all life on Earth.”* With its holistic perspective on health in relation to natural and social environments, Planetary Health distinguishes itself from the traditional discipline of *environmental health*. The latter emerged in the second part of the twentieth century and focuses on local exposures and conditions (ex: chemical agent, extreme temperatures) that negatively impact human health [(NEHA)^1^; (25)]. Offering a more comprehensive approach to health in its relation to the Earth's natural systems, a Planetary Health perspective appears better adapted to face the unprecedented synergistic challenges of environmental and public health that humanity faces, as it stresses that health is determined by the functioning of the Earth's system. Furthermore, the Planetary Health principles are aligned with the United Nation (UN) Sustainable Development Goals (SDGs) when taken in a systemic perspective: good health and wellbeing for all (SDG 3) need to be considered in relations to the other psychological, socio-cultural and economic goals, all embedded within the limits of the biosphere (SDGs 6, 13–15) (26, 27). However, the fact that such important determinants of health is undermined by the current ecological and climate emergencies (22) is still not translated into public policies commensurate with the challenges ahead.

Numerous recent position papers have called for a widespread recognition of Planetary Health objectives and the ethical obligation of healthcare professionals to concern themselves with the political and economic structures leading to planetary damage, including via public health interventions on such systemic factors (16, 18, 28–35). The narrowest application of Planetary Health's principles to clinical care and healthcare services seeks to mitigate the associated greenhouse gas (GHG) and other pollutants emissions. A broader application could instead question the structure, content and objectives of healthcare services themselves, as a mean of producing better healthcare practices and improving population health.

While useful at promoting an ambitious global public health discourse, these calls have nonetheless remained mostly theoretical and without questioning the goals to attain for human health in terms of traditional health measures. Little research on practical applications of Planetary Health and considerations for local contexts in the healthcare sector exists to date (36–38). As we face increasing threats from global anthropogenic environmental degradations, a major challenge will be to translate global concepts such as Planetary Health to healthcare provision and incorporate it in the health profession deontology at local level (39). Although the impact of these systemic environmental degradations are global, they are indeed experienced locally through individual and community health and wellbeing. The responses (adaptation to new health treats) and preventive actions (mitigation of environmental degradations) will have to be locally implemented, though supported by national or supranational political bodies (40). In this context, it is imperative to explore ways to integrate the Planetary Health concept into concrete and locally anchored health services and practices.

### Challenges for Healthcare Services and Biomedicalization of Health

Current Western healthcare systems are facing their limits when it comes to providing affordable, equitable, and sustainable healthcare services (41–48). Epidemiological transitions (moving from predominant infectious diseases to chronic diseases), demographic trends (aging of the population) and “biomedicalization” of healthcare of the last decades (treating all health problems through a biomedicine approach) have placed healthcare systems worldwide under economic pressure and are now challenging the very care of individuals and populations (49).

In theory, contemporary clinical practices and healthcare services strongly value patient-centered approaches and partnerships in the frame of participatory approaches (e.g., shared decision making) (50). These promises to meet individual's health needs in a comprehensive perspective are based, among other things, on the widely known “biopsychosocial model” developed by Engel in the late 70's (51, 52), as well as on the long-standing discourses of empowerment in health promotion and education ((53, 54)). These theoretical and clinical “holistic” approaches have strongly influenced the training and practice of physicians and healthcare professionals on one side, while being regularly criticized on the other (see below). A question remains to be settled, instead, as to the level of influence these models have had on practice and organization of the healthcare system. While these provide an integrative vision of the biological, psychological and social determinants of health and illness, implementations have been lacking or proving complicated in a context characterized by an increasing normative standardization of care through the evidence-based medicine paradigm (e.g., guidelines, checklists, quality indicators, etc.) and a high degree of specialization of healthcare services. These developments, which result in a fragmented organization of healthcare services between sectors and within disciplines, hinder the consideration of the complex interactions between the different dimensions of health and limit the applicability of complex bio-psycho-social as well as participatory models of healthcare. More generally, maybe due the somehow vague concept of holism (even if it is probably better defined in nursing sciences than in medicine), such a model also contains limitations in itself. Importantly, it fails to consider that health is also dependent on the ecological functioning of the Earth's ecosystems to provide favorable living conditions for humans and non-humans.

Furthermore, healthcare services are currently mostly oriented on acute, biomedical and hospital care, where most financial resources are used (16, 42–48, 55–62). Health policies focus on care delivery, meaning the health system focusses on the same structure. This orientation means that prevention and non-medical determinants of health are largely neglected (59, 63–70), even though biomedically actionable determinants of health are estimated to account for only one fifth of the health of populations and individuals (71–73).

Even those biomedical approaches that dissect the biological effects of environmental exposures (e.g., epigenetics, exposomics) often retain a reductionist allure that shies away from a complex uptake of the mixed bio-socio-environmental dynamics producing health in research and policy (74–76). This is not just a matter of restructuration and adapting the organization of care but instead deeply questions the epistemological conceptualization of health. In fact, health policies' and interventions often focus on biomedical and individual responsibility for health—thus masking the patterning of health behaviors by unequally distributed social and material circumstances and environmental exposures and the need to address them in order to tackle health inequalities in the population (32, 77–80).

It is also worth noting that the economic logics that govern current healthcare services (i.e., productivity and profit-oriented) share similarities with the predominant neoliberal organization of society. And that a consumerist logic both encourages unhealthy lifestyles and lead to the current planetary environmental emergency (58, 81–83). These epistemological, organizational, technological and policy factors converge to produce healthcare services insufficiently oriented toward community, public health and primary Care (84–90). Yet, as highlighted by WHO (90) and decades of research on improving healthcare systems (91), primary care, in close collaboration with the public health sector, is the one that will have to shoulder the bulk of the health challenges in the future.

Finally, in recent years a number of reports have highlighted the non-negligible detrimental environmental impacts of healthcare services, including the emissions of greenhouse gas (GHG) and the impacts of pharmaceuticals and other pollutants released in the environment (43, 92, 93). Hospitals carry out the large majority of this burden (94), which further supports the call for a reorganization of healthcare services.

The current configuration of healthcare systems, which largely determines the kind of care delivered, is therefore not sustainable from many perspectives: demographic, economic and ecological. It is thus essential to fundamentally rethink health as well as healthcare services from this tripartite perspective.

### General Hypothesis: Focusing on Primary Care to Integrate Planetary Health Principles

In light of the growing threats for human health stemming from anthropogenic environmental degradations (6) and the limits of the current healthcare systems, this article hypothesizes that the Planetary Health perspective should be used to rethink how we define health and to redesign the organization of healthcare services, identifying new roles and activities for primary care actors, by better integrating socio-environmental determinants of health. We furthermore argue for the need of greater public engagement and advocacy from healthcare professionals, in coordination with other sectors, to see that socio-economic structures provide favorable living conditions for today's and future generations This is in line with calls for greater public engagement from universities and teaching institutions in a time of climate and ecological emergencies (95).

Health authorities necessarily need to find an equilibrium between society's expectation in terms of “being in good health” and the availability of resources on the other. If it is already widely recognized that sustainability of healthcare systems is undermined by financial limitations, with costs particularly driven up by constant medical technological innovations and population aging, the growing threats posited by worldwide environmental degradations call for a greater recognition of the ecological limits of the Earth's natural system. However, we argue that these considerations should not lead to limit or reduce health and wellbeing of population. Quite the contrary, the adoption of Planetary Health principles as a “strategic plan” to address a number of systemic issues of healthcare services could contribute to better and more sustainable healthcare services for the populations. However, a strong transdisciplinary approach rooted in the local context of communities is essential to meet this challenge.

### Propositions

We make two propositions that can help to integrate and translate the global and abstract principles of planetary health to a local level of redefined health services. The first proposition relates to a theoretical contextualization and conceptualization of health. The second “implementation” proposition defines the practical integration of Planetary Health into primary care.

## Framing a New Approach to Health

### The Limits of Current Definitions of Health

The idea that our bodies and our health are entangled with their socio-material environment extends back to several centuries ago. While the use of the term “environment” mostly dates back to the Nineteenth century in various languages to describe what surrounds a living being, and its link to ecological thinking is a product of Twentieth century ecological and natural sciences, views of the body and its alterations resulting from environmental changes are part of pre-to-modern medicine since Hippocrates' “Airs, Waters, and Places”. By considering pathologies as the expressions of imbalances of the organism in interaction with its environment, Hippocratic medicine paid specific attention to the interrelationship of human beings to their environment, and to the ways in which this articulation is continuous and undone throughout individual or collective history (96–100). If it is difficult to propose a unique definition of health, it is widely acknowledged that part of it integrates the subjective experience of wellbeing that includes the satisfaction of physical, cultural, psychosocial, economic and spiritual needs, fundamental to individuals as well as to families and communities (101). Similarly, nursing sciences have developed long standing theories considering that it is in its essence to treat the whole patient in its care: mind, body, spirit, embedded in its surrounding environment (102).

However, if it is also clear from a theoretical point of view that systemic relationships exist between social, economic and environmental health determinants, the practice of modern medicine and healthcare in general has largely separated and fragmented theses domains (102). This led to the paradoxical tendency to treat most health problems as bio-medical conditions on one hand, and to insufficiently consider environmental or social issues as health problems on the other despite longstanding existing evidence from social epidemiology (48, 103). The Planetary Health agenda applied to primary care therefore needs to draw on the long-standing recognition of the interplay between health and the environment in philosophy and social sciences, then to strengthen the recognition of the dependence of health from natural ecosystems.

In this context, “what is being healthy?” is a question that needs to be carefully examined and rethought. The answer to this question should go beyond the 1946 WHO definition (“Health is a state of complete physical, mental and social wellbeing and not merely the absence of disease or infirmity”). It has been indeed criticized as being unachievable (what does “complete wellbeing” means?”), written at a time when acute diseases were predominant and difficult to operationalize (104, 105). Other definitions of health include the one by the philosopher Marie Gaille (99) who argues for “*the need to apprehend health as a dynamic state in which the individual is able to organize a world and act in a meaningful way for him/her*” (translation from French by the authors). This definition, which focuses on the individual's experience rather than its biomedical condition, could serve as a basis for further developments. However, most definitions are still limited to explicitly account for the dependence of human health on the integrity of the Earth's ecosystems. Do we live a healthy life if we contribute, voluntarily of not, to the worldwide anthropogenic degradations that undermine the functioning of the Earth's life sustaining systems? Ultimately, this reveals a new problem related to the temporality of health. Indeed, it refers to the idea of “future health” as global present anthropogenic threats put at risks health of human beings in the future (106).

These theoretical considerations must be the subject of a social debate before being “operationalized” at clinical level with patients in daily practices. In order words: “how does this new perspective of health match individuals' (and patients') personal health & wellbeing priorities?” This question is important, the answer not obvious and it raises numerous ethical issues but must be faced collectively in order to live well and healthily within limits.

### Enhancing the Biopsychosocial Models

Some of the critiques of the biopsychosocial models of health have unfairly portrayed this family of models as either an idealist slogan providing little help to clinical practice (107–109), or as an instrumental rhetoric to promote the “wolf” of biomedicine in “sheep” holistic clothing. But we argue that the biopsychosocial model can still be useful to instruct a working definition of health, clinical teaching and activities in a Planetary Health perspective. First, this complex psychosocial thinking about health currently regains centrality as a symmetric approach to biomedicine. Toxicological research on the social, psychological and behavioral modulations of the epigenetic and exposomic effects of chemicals ((110)) epitomizes the growing importance of such an all-encompassing vision of health. It is simply an artifact of academic traditions and disciplinary divisions to separate the organic and the relational, the biological and the social determinants of health. If our behaviors, which are socially patterned, modulate the biological (e.g., epigenetic) effects of exposures to chemicals, then should the challenge rather be an integrative scientific approach to the study of all these factors in combination? (111). Secondly, the urgency of such integrative biopsychosocial science, straddling the life and social sciences, offers the opportunity to integrate natural environmental dimensions, as well as political and economic structures that lead to planetary damage. Sticking to the example above, the psychological and social modulators of toxicity cannot be understood without taking into account uneven geographical distributions of substances, historical legacies of pollution, or even the documentation of health promotion practices toward environmental protection and consciousness in a given setting. In clinical practice, it is common to explore different kind of healthy or unhealthy exposures such as smoking or doing dangerous sports. But in order to fully integrate Planetary health principles, it should be important to explore also exposures related to the natural environment such as the level of biodiversity that patients are exposed to. Indeed, while we observe a global decline in biodiversity, an emerging field of research points to the role that biodiversity plays in the regulation of the human immune system. Numerous studies link reduced exposure to microbial biodiversity, reduced diversity or imbalance of the human microbiota, to the increasing prevalence of allergies and chronic inflammatory diseases in urban populations around the world. But in order to fully integrate Planetary Health principles, it should be important to also explore additional types of exposures related to the natural environment. For example, numerous studies showed that a reduced exposure to microbial biodiversity leads to a reduce diversity or imbalance of the human microbiota what increase the prevalence of allergies and chronic inflammatory diseases in urban populations around the world (112–114). Thus, healthcare professionals should not only treat these issues, but should concern themselves about the global and local decline of biodiversity and reduced opportunities of contact with natural and biodiverse greenspaces for urban populations. This topic needs to be addressed with patients and healthcare professionals should also advocate at the community level for greener and more biodiverse cities.

## Focusing on Primary Care to Integrate Planetary Health Principles

### Primary Care and Public Health Interplay

Public health interventions have proved to be a crucial tool to protect populations from environmental hazards (115). Examples of public health initiatives to tackle environmental health issues include reducing indoor and outdoor air pollution, improving sanitation and drinking water and protection against climate-related hazards such as heat or heatwaves. However, the effectiveness of these initiatives is sometimes limited by a lack of participation, poor policy commitment, limited resources, or limited trans-sectorial collaborations. Furthermore, in light of the unprecedented coupled environmental and public health challenges humanity is facing, public health needs to evolve to adapt a more integrative and systemic perspective of the relations between health and natural and social environments, strengthening collaborations with institutions and departments involved in sustainability strategies. There is indeed a real need to identify the overlap of their respective objectives, the specific or transversal levers of the different fields and how they could collaborate to reinforce their respective interventions.

Primary care itself should accompany these changes by adopting new roles and activities. Indeed, with its holistic, interdisciplinary and longitudinal approach to patients, strongly enrooted in the community, it also represents a key setting that needs to transform its approach to health, with a greater integration of socio-environmental determinants and an ecosystemic perspective of health and wellbeing. All the more so, if we consider that the primary care is used to taking care of patients whose health is affected by multiple determinants of health (i.e., individual behaviors, social context, biological specificities). Furthermore, while it is primarily accustomed to the clinical care of individual patients, we argue that primary care needs to recognize public health activities at the community level as a constitutive part of its role in society, anchoring larger public health programs developed at the regional or national level (116). This local level of population care is indeed often deficient in Western societies, but is essential to improve the health of populations through interventions that are meaningful to citizen in their local context. In that perspective and in light of the sanitary impacts predicted to result from the current ecological and climatic emergencies, primary care and public health should strive to accompany transformative policies needed to decarbonize our societies, in particular when these environmental policies offer direct health co-benefits. Health cobenefits are well referenced for transports policies or the promotion of more sustainable diets (117).

Developing a population responsibility in primary care on health (118) could improve population's health and reduce environmental degradations' burden, while decreasing the overall financial and human resources needed to run current bio-medical-oriented healthcare systems. A continuum, from individuals to populations, is necessary to implement effectively a systemic perspective on health and health interventions as advocated by Planetary Health but this integrative approach of public health and primary care remains insufficiently applied (116). Last but not least, this should go in parallel with a deep rethinking of the approach to health problems that integrate non-medical interventions and better consider collaborations with social and community sectors. Indeed, there is nowadays a trend to see all health problems through a biomedical lens, which is obviously not desirable and inefficient (48, 59). In this sense, the integration of a Planetary Health perspective in primary care settings effectively argues for better integration of socio-environmental determinants of health, and thus transforming the content of medical consultations by expanding its fields of activity beyond the current bio-medical domains. This will require the development of new models of care that valorize interprofessional and non-medical collaborations.

### Integrating a Planetary Environmental Perspective Into Clinical Practice and Primary Care

The World Organization of Family Doctors (WONCA) has called for family doctors to realize the relevance of Planetary Health principles for their clinical practice. But concrete novel interventions, implemented in the clinical setting, that have been assessed for their feasibility, acceptability and effectiveness remain scarce. The WONCA for example encourages clinicians to advise patients about important “co-benefits” of changes in their daily choices and habits, that can both benefit their own health and reduce their environmental impact (35). Healthcare professionals could for example encourage their patients to walk or cycle more for their daily commute instead of using fossil-fueled transportation, thus providing direct health benefits for themselves through physical activity, population health benefits through reduced air pollution and environmental co-benefits through reduced CO_2_ and other pollutant emissions. If convincing evidence shows that cycling benefits one's health and that of the environment, recommendations are needed for how GPs could encourage their patients to change their behavior with this dual argument in mind. In addition, the acceptability and effectiveness of such a motion will be highly influenced by structural conditions such as the presence of safe and efficient cycling and walking infrastructure on the patient's commute and the patient's personal experience with cycling. Work by Nelson can be cited as one of the few examples that attempted to conceptualize clinical ecology in an holistic approach, albeit theoretically (33). Some recent initiatives in the UK encourage “green social prescribing,” referring to nature-based activities such as gardening or outdoor walking activities offered to patients in the community and aiming to improve mental health outcomes, reduce health inequities and reduce demand on the health and social care system (NHS.UK). But further research and experimentation in this field is clearly needed. A number of initiatives such as *healthcare without arm* (https://noharm.org/) or *green doctors* in the UK offer some concrete propositions to encourage greener clinical practice.

### Engagement at the Community Level

The responsibility of health professionals in raising awareness on the health impacts of climate change and other ecological emergencies is increasingly recognized (57, 119). Benefiting from their image of holders of legitimized medical knowledge, health professionals should not only discuss these issues with their patients (29, 120), but also use their health expertise and community embeddedness to influence environmental policies (121, 122). For example, if healthcare professional encourage patients to walk or cycle more for their daily commutes, one could argue that they should also advocate at the community level for an urban planning that favors such forms of mobility. In this way, they could support measures resulting from climate strategies and facilitate their implementation. As stressed in the WONCA declaration, there is a role for health professionals to advocate in their practice and professional groups for the recognition and the transformation of structural factors that drive individual behaviors and negatively affect health. A bit like John Snow (a general practitioner) who, observing his patients dying from cholera in Soho, London, in 1854, advocated for the shutting down of the water pump that he thought responsible for the infections. While this role may be played by some health professionals already, based on their own initiatives, there is a need to promote it more globally and to make it part of their medical responsibility.

## Conclusions

In recent years, environmental issues have imposed themselves on the political agenda but the discourse has often not given way to measures commensurate with the stakes involved. It is increasingly recognized that tackling these complex issues will require integrated and interdisciplinary approaches, taking place at different levels and involving different actors and stakeholders (26, 123, 124). If health services will have to adapt to the sanitary impacts of environmental degradations, they should also ambition to contribute and accompany the societal transformations required to respect Planetary boundaries. This perspective calls for a greater recognition of the links between human health and ecosystems integrity, and accordingly integrate these considerations in the organization and provision of healthcare services and clinical practices. This is even more important as current healthcare services are reaching their limit in providing affordable, equitable and decent care to the population.

For this, we propose to develop a pragmatic research agenda that is able to integrate to primary care Planetary Health principles through two main propositions:

**(1) Theoretical:** Redefining health in order to better integrate its dependence on natural environments and enhancing the biopsychosocial model to a “biopsychosocial and environmental model.”
**(2) Implementation:** Redesigning healthcare services by focusing on primary care because of its strong link with the community and transforming its organization, creating new models of care that better integrate public health, social, environmental and community sectors, while transforming medical services by adopting new roles and activities that integrate socio-environmental determinants of health.

To accompany this change of paradigm, it is of prime importance to develop implementation research in this new field, as we observe that many initiatives are developed (such as green prescribing) without being based on clear effectiveness evidence. And last but not least, the implication of health economists would be crucial to the development of sustainable healthcare models and should aim to explore how to extend the notion of “value” by systematically considering environmental and social constraints and impacts in resource allocation. In order to ensure an intersectoral coherence, ecological economics and policy analysis should examine how public health and healthcare work are conceptualized, prioritized and implemented in the proposed environmental policy packages currently put forward at the regional, national and international levels around the world. This research must be anchored in the community, involve public institutions, citizens and healthcare practitioners such as to offer opportunities for concrete actions. For example, it might be of interest to conduct pilot projects that integrate recommendations for clinicians on how to integrate co-benefits approaches (for mobility or diet for example), adopt eco-directed prescription or nature-based therapies. This could be coupled with payment schemes that encourage health professionals' change of practice. And last but not least, this should be accompanied by a thorough evaluation of the impact on both patients and the environment.

## Data Availability Statement

The original contributions presented in the study are included in the article/supplementary material, further inquiries can be directed to the corresponding authors.

## Author Contributions

NS and JG-H wrote the first draft of the manuscript and led the conception of the project. All other authors contributed equally to the scientific development of the project and contributed to the manuscript for their respective discipline and approved the submitted version.

## Funding

The study was internally funded by the authors. Open access funding provided by University of Lausanne.

## Conflict of Interest

The authors declare that the research was conducted in the absence of any commercial or financial relationships that could be construed as a potential conflict of interest.

## Publisher's Note

All claims expressed in this article are solely those of the authors and do not necessarily represent those of their affiliated organizations, or those of the publisher, the editors and the reviewers. Any product that may be evaluated in this article, or claim that may be made by its manufacturer, is not guaranteed or endorsed by the publisher.

## Footnotes

^1^NEHA. Available online at: www.neha.org/about-neha/definitions- environmental-health.
