# Corrigendum: Translating planetary health principles into sustainable primary care services

**DOI:** 10.3389/fpubh.2022.1075712

**Published:** 2022-11-08

**Authors:** Julia Gonzalez-Holguera, Marie Gaille, Maria del Rio Carral, Julia Steinberger, Joachim Marti, Nolwenn Bühler, Alain Kaufmann, Luca Chiapperino, Ana M. Vicedo-Cabrera, Joelle Schwarz, Anneliese Depoux, Francesco Panese, Nathalie Chèvre, Nicolas Senn

**Affiliations:** ^1^Competence Centre in Sustainability, University of Lausanne, Lausanne, Switzerland; ^2^Laboratory SPHERE, UMR 7219, University Paris Diderot CNRS, Paris, France; ^3^Institute of Psychology, University of Lausanne, Lausanne, Switzerland; ^4^Institute of Geography and Sustainability, University of Lausanne, Lausanne, Switzerland; ^5^Department of Epidemiology and Health Systems, University Center for Primary Care and Public Health (Unisanté), Lausanne, Switzerland; ^6^STS Lab, Institute of Social Sciences, University of Lausanne, Lausanne, Switzerland; ^7^ColLaboratoire (ColLAB), University of Lausanne, Lausanne, Switzerland; ^8^Institute of Social and Preventive Medicine (ISPM), University of Bern, Bern, Switzerland; ^9^Oeschger Center for Climate Change Research, University of Bern, Bern, Switzerland; ^10^Department of Family Medicine, University Center for Primary Care and Public Health (Unisanté), Lausanne, Switzerland; ^11^Centre Virchow-Villermé and Centre des Politiques de la Terre, Université Paris Cité, Paris, France; ^12^Institute of Social Sciences, University of Lausanne, Lausanne, Switzerland; ^13^Faculty of Geosciences and the Environment, Institute of Earth Surface Dynamics (IDYST), University of Lausanne, Lausanne, Switzerland

**Keywords:** planetary health, primary care, health services, health professionals, interdisciplinary, sustainability

In the published article, there are several errors in the affiliations of the authors. We apologize for these mistakes that we didn't notice, but it seems that between the initial submission and the proofed article, changes occurred probably due to the system (they were correct in the manuscript), also considering that almost all affiliations are mixed-up.

The authors apologize for this error and state that this does not change the scientific conclusions of the article in any way. It is also to be noted that the list of authors has not changed, neither their respective contributions.

We would be grateful if you could make these important changes.

- For **Joachim Marti**, Instead of “^5^ Department of Family Medicine, Center for Public Health and Primary Care, Lausanne, Switzerland.”
- The correct affiliation is “^5^ Department of Epidemiology and Health Systems, Center for public health and primary Care, Lausanne, Switzerland.”
- For **Ana Maria Vicedo-Cabrera**, Instead of “^8^ Institute of Social Sciences, University of Lausanne, Lausanne, Switzerland, ^9^ Institute of Earth Surface Dynamics (IDYST), University of Lausanne, Lausanne, Switzerland.”
- The correct affiliations are “^8^ Institute of Social and Preventive Medicine (ISPM), University of Bern, Bern, Switzerland, ^9^ Oeschger Center for Climate Change Research, University of Bern, Bern, Switzerland.” Also, please write the name of the author Ana M. Vicedo-Cabrera.
- For **Joelle Schwarz**, instead of “^5^ Department of Family Medicine, Center for Public Health and Primary Care, Lausanne, Switzerland.”
- The correct affiliation is “^10^ Department of Family Medicine, Center for Public Health and Primary Care, Lausanne, Switzerland.”
- For **Anneliese Depoux**, instead of “^10^ Institute of Social and Preventive Medicine (ISPM), University of Bern, Bern, Switzerland.”
- The correct affiliation is “^11^ Centre Virchow-Villermé and Centre des Politiques de la Terre, Université Paris Cité, Paris, France.”
- For **Francesco Panese**, instead of “^11^ Centre Virchow-Villermé and Centre des Politiques de la Terre, Université Paris Cité, Paris, France.”
- The correct affiliation is “^12^ Institute of Social Sciences, University of Lausanne, Lausanne, Switzerland.”
- For **Nathalie Chèvre**, instead of “^7^ColLaboratoire (ColLAB), University of Lausanne, Lausanne, Switzerland.”
- The correct affiliation is “^13^ Institute of Earth Surface Dynamics (IDYST), Faculty of Geosciences and the Environment, University of Lausanne, Lausanne, Switzerland.”
- For **Nicolas Senn**, instead of “^5^ Department of Family Medicine, Center for Public Health and Primary Care, Lausanne, Switzerland.”
- The correct affiliation is “^10^ Department of Family Medicine, Center for Public Health and Primary Care, Lausanne, Switzerland.”

The only additional affiliation is for Ana M. Vicedo-Carbera as mentioned above “^9^Oeschger Center for Climate Change Research, University of Bern, Bern, Switzerland.”

The authors apologize for this error and state that this does not change the scientific conclusions of the article in any way. The original article has been updated.

## Publisher's note

All claims expressed in this article are solely those of the authors and do not necessarily represent those of their affiliated organizations, or those of the publisher, the editors and the reviewers. Any product that may be evaluated in this article, or claim that may be made by its manufacturer, is not guaranteed or endorsed by the publisher.

